# Ageing well in a foreign land as a process of successful social identity change

**DOI:** 10.1080/17482631.2018.1508198

**Published:** 2018-08-13

**Authors:** Jolanda Jetten, Sharon Dane, Elyse Williams, Shuang Liu, Catherine Haslam, Cindy Gallois, Vivienne McDonald

**Affiliations:** a School of Psychology, University of Queensland, Queensland, Australia; b School of Communication and Arts, University of Queensland, Queensland, Australia; c Faculty of Health and Social Sciences, University of Queensland, Queensland, Australia; d Diversicare-ECCQ, Brisbane, Australia

**Keywords:** Healthy ageing, social identity change, migration, adaptation, migration experiences

## Abstract

**Purpose:** Over and above the risks associated with ageing, older migrants are also at risk of social isolation. The social identity approach, and the Social Identity Model of Identity Change (SIMIC) in particular, provides a theoretical basis from which to understand the factors contributing to social isolation and how this then impacts on older migrants’ capacity to age well in a foreign land. Building on the recognition that migration involves a major life change, we explore this transition qualitatively focusing specifically on social connectedness and adjustment.

**Methods:** In semi-structured interviews with 29 older migrants in Australia, we examined participants’ experiences of migration and perceptions of identity and identity change. We also considered in more detail experiences of the most and least socially isolated individuals to understand adjustment trajectories.

**Results:** We found evidence supporting the key processes described in SIMIC (relating to social identity continuity, social identity gain, and perceived identity compatibility), suggesting that where adjustment was positive it was experienced as a process of successfully adapting to identity change.

**Conclusion:** We emphasise the importance of identity resources as substantial and concrete assets that can enhance the well-being among older adults aging in a foreign land.

Migration is often a challenging experience because new opportunities and new life experiences go hand in hand with difficulties associated with cross-cultural adaptation and exclusion (Badea, Jetten, Iyer, & Er-rafiy, 2011). These challenges are magnified for older migrants, who not only have to adjust to the changes that ageing itself imposes, but do so in a country that is foreign. When we consider these dual changes to which people must adjust, it is not surprising that the well-being of older migrants is particularly vulnerable and in many cases poor. For example, Abbott and colleagues (Abbott et al., 2003) found in New Zealand that one in four older Chinese immigrants reported depressive symptomology. Moreover, Yu (1986) found that suicide rates among Chinese older migrants in the USA were much higher than among older non-migrant Americans. In a qualitative study, Greek older migrants living in Australia described ageing as a particularly disempowering and challenging experience, largely due to the perceived lack of family support (Walker, Newman, Tsianikas, Panagiotopoulos, & Hurley, 2013).

While there are a number of factors that contribute to these poor health outcomes, researchers have identified social isolation as a key contributor. Across a range of populations, social isolation has been shown to be associated with multiple indicators of poor well-being: it is correlated with reduced self-esteem (Jetten et al., 2015), increased depression (Haslam et al., 2010), low psychological well-being (Liu, 2011), and increased mortality (House, 2001). Because of this, researchers have argued that older migrants face a double hit to their well-being—over and above the challenges associated with ageing, older migrants are particularly prone to social isolation (Abbott et al., 2003). Moreover, social disconnection affects not only those migrants who moved countries at an older age, but also those who moved earlier in life. So there are clearly two challenges that people face in ageing well in a foreign land: losing valued relationships with people from their home country and trying to establish new relationships with people in their host country.

A question that presents itself is why some migrants experience greater socially isolation following migration. Attempting to identify the factors that contribute to social isolation and reduced well-being, researchers have pointed to the lack of social and emotional support that migrants experience in their new home country (Abbott et al., 2003; Park & Kim, 2013). In particular, aside from barriers to participation associated with culture and language (Ip, Lui, & Chui, 2007; Westcott & Vazquez Maggio, 2015), it has been suggested that poor health and limitations in mobility and independence make it difficult for older migrants to shape their life in ways they wish (Meijering & Lager, 2014). As a result, even among those who have lived in the host country for many years, older migrants may still have less access to social support networks compared with their older non-migrant counterparts (Park & Kim, 2013). As a result, they perceive a lack of control, and this may lead to apathy and an inability to make use of available social support (Walker et al., 2013). All these challenges lead to acculturative stress, which is manifested in increased levels of depression, reduced self-esteem, lower perceived quality of life, and health problems (Kim, Heo, & Park, 2014).

A number of theories have been used to explain poor well-being and adjustment to the host culture, many of which focus on relationships between acculturation and health. More positive health outcomes are typically seen when people can both maintain a connection with their culture of origin and adopt aspects of the new culture (Abbott et al., 2003; Berry, 1997; Williams & Berry, 1991). Indeed, meta-analytical evidence from 83 studies with more than 20,000 participants has shown that integration (i.e., holding on to both cultures) is associated with better well-being outcomes and socio-cultural adaptation (Nguyen & Benet-Martinez, 2013). However, for the reasons suggested above, integration is not easy for many older adults. Although these might apply to migrants of all ages, their impact tends to be stronger among older migrants, particularly where they become more dependent on other people in their later years (Meijering & Lager, 2014). The result is that some older migrants feel that they are neither socially connected to where they come from, nor where they are residing (Liu, Volcic, & Gallois, 2015). This has obvious consequences for social isolation and, in turn, well-being.

Building on a growing body of work that demonstrates the importance of social connectedness for health (for an overview see Haslam, Jetten, Cruwys, Dingle, & Haslam, 2018; Jetten, Haslam, Haslam, Dingle, & Jones, 2014), this study explores themes of social connectedness and adjustment among older migrants in Australia. In particular, guided by the Social Identity Model of Identity Change (SIMIC, Haslam et al., 2008; Jetten, Haslam, Haslam, & Branscombe, 2009), we explore whether migration experiences can be understood through the lens of social identity change. This model proposes that ageing in a foreign land is associated with a sense of *identity loss*, and that overcoming such loss to successfully transition and adapt to life change requires some form of identity reconstruction. In this study we draw on people’s accounts of their migration experiences to determine the extent to which themes relating to these processes of social identity continuity, social identity gain and identity compatibility are part of the adjustment process.

## The social identity model of identity change (SIMIC)

SIMIC builds in important ways on the social identity approach—which consists of social identity theory (Tajfel & Turner, 1979) and self-categorization theory (Turner, Hogg, Oakes, Reicher, & Wetherell, 1987). Central to the social identity approach is the idea that social groups (whether they be family, interest, community, or other groups) are an important basis for self-definition, such that the more one identifies with the group, the more influential that group is in informing a person’s thoughts, values, and behaviour. Such identification provides people with a sense of grounding and belonging (Jetten et al., 2015). In the context of ageing in a foreign land (particularly in a foreign-language community), such connectedness can be extremely beneficial, because group memberships—and the social identities that are derived from them—provide access to psychological resources providing people with a sense of belonging and connection, support, meaning, and a greater sense of personal control (Cruwys, Haslam, Dingle, Haslam, & Jetten, 2014; Greenaway, Cruwys, Haslam, & Jetten, 2015; Haslam et al., 2018; Jetten et al., 2014). These are the resources that people draw on to cope with challenges brought about by the need to abandon old pre-migration group memberships and connections (and the social identities associated with them) and the need to take on new social identities through joining new groups in the post-migration context. That is, the challenge of identity change.

**Figure 1.**
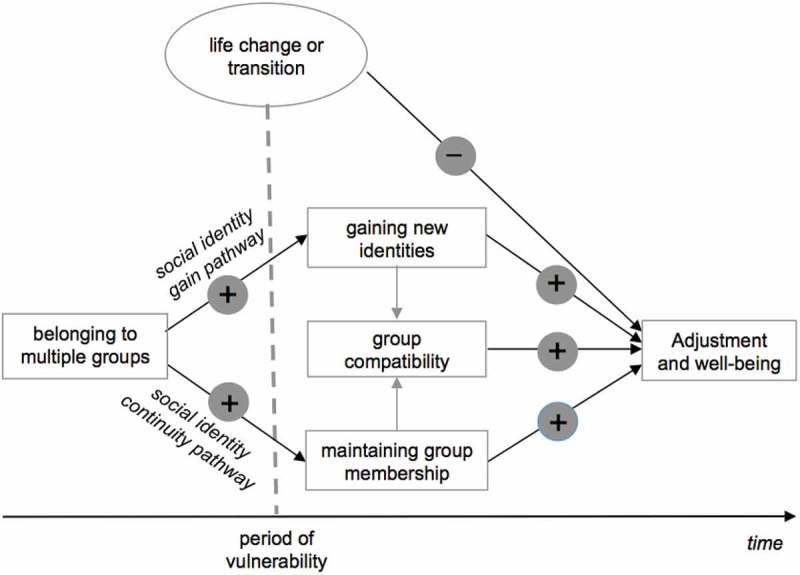
SIMIC identifies two key pathways that help people cope with identity change in the face of major life transitions and events. The first is a *social identity gain* pathway associated with the acquisition of new group memberships; the second is a *social identity continuity* pathway associated with the maintenance of pre-existing group memberships. Both pathways are more likely to be accessible the more group memberships a person had prior to the life transition. The impact of the two pathways on health and well-being also depends on the compatibility of the social identities they involve. Figure adapted from Haslam et al. (2018).

However, because social identities are central to successfully adjusting to life changes, they are also the key reason why such changes are psychologically challenging. Precisely because group memberships provide essential psychological resources, when these social identities are altered or lost through migration, individuals’ capacities to adjust are jeopardized and well-being can be negatively affected as a result. SIMIC proposes two pathways through which successful adjustment to life change, such as migration, is supported: (a) maintaining connections with some existing social groups, to provide a sense of social identity continuity, and (b) joining new social groups to provide a basis for social identity gain (see Figure 1). Furthermore, these two pathways are more likely to be viable routes (and thus protective of well-being during change) when a person belongs to more social groups and has a richer social network when migrating.

The model also suggests that the compatibility of pre-migration and post-migration identities further facilitates or blocks the extent to which individuals experience social identity continuity and/or social identity gain. We elaborate on these pathways below before providing a rationale for the present study.

### Social identity continuity

SIMIC proposes that people find comfort in the knowledge that their past and present are connected (Jetten & Hutchison, 2011; Jetten & Wohl, 2012; Sani et al., 2007) and there is evidence that social identity continuity is important when facing important life transitions. For example, among people who had suffered a stroke, it has been shown that maintaining pre-stroke group memberships enhanced life satisfaction to support their recovery post-stroke (Haslam et al., 2008). Further evidence showing the importance of continuity comes from clinical populations who experience discontinuity. Clinical research shows that perceived disconnection between the past and present is associated with negative affect, anxiety, alienation, depression, and even suicide (e.g., Bluck & Alea, 2008; Chandler & Proulx, 2008; Lampinen, Odegard, & Leding, 2004; Pennebaker, 2003). Not surprisingly, therefore, people are motivated to achieve and maintain a sense that their past and present are connected, and one way to achieve that is by maintaining a sense of belonging to “old” groups and social networks when encountering life-changing events.

This notion of social identity continuity is clearly relevant to the migration context. Moving countries not only influences cultural connectedness but also social connectedness, because migration requires people to move away from old group memberships and the social identities that these entail. In this process, old social identities can become less important and are often lost. The perception of being disconnected from valued social identities in the country of origin may reduce a person’s social connectedness in the transition, dwindling their psychological resources (of support, perceived control, etc.) when they need them most (Iyer, Jetten, & Tsivrikos, 2008; Sani, 2010).

The acculturation literature (e.g., Berry, 1997) speaks to the theme of social identity loss, and there is evidence that activities that restore social identity continuity enhance adjustment in the new context. It has been found that participation in ethno-cultural activities (e.g., preparing food with others from the country of origin) is particularly important to maintain a sense of social identity continuity, because it not only provides a sense of social connectedness but also helps to extend the cultural heritage to the new cultural context (Liu et al., 2015). By extension, this can strengthen the link that people have with their “roots” ( Sani, 2010). Recent research on older migrants in the Netherlands shows that feeling connected with one’s roots allowed migrants to experience a sense of belonging, and where this occurred, it was associated with enhanced well-being (Meijering & Lager, 2014).

### Social identity gain

SIMIC further proposes that negative effects of identity change on well-being can be reduced to the extent that people take on new identities and join new groups after the life transition. There is some evidence in a range of contexts, varying from coming to terms with acquired brain injury (Jones et al., 2012) to entering university (Iyer, Jetten, Tsivrikos, Postmes, & Haslam, 2009), that the negative consequences of identity change are attenuated when people readily take on new social identities or join new groups. This is because social identities derived from group memberships can provide a basis for the provision and receipt of social support and help people to reorient to new situations and contexts (Iyer et al., 2009).

We propose that joining new groups and taking on new identities may also be beneficial for a person’s adjustment to migration. In particular, connecting with other migrants may have positive consequences for well-being because, aside from the social support that is provided by new groups in challenging times, knowledge of other people sharing similar experiences (e.g., ageing in a foreign land) creates a common ground which can serve to expand the sense of self beyond one place or culture (Liu et al., 2015). Taking part in the activities of groups other than one’s ethnic group, in particular, has been found to be associated with better sociocultural adjustment as opportunities to interact with people beyond the home culture can enable migrants to develop skills that help them to adjust to the changed cultural environment (Ward, 2013). Consistent with this reasoning, there is not only evidence that migrants often quickly rebuild their social connections (and thus their social support base) after migrating (Butt & Moriarty, 2004; Kim et al., 2014), such enhanced social connectedness has also been found to be beneficial for the well-being of older migrants (e.g., Cook, 2010; Meijering & Lager, 2014).

### Social identity compatibility

Critically, however, it is not the case that just any social connection will do. For new groups to provide a positive basis for support, it helps that they are ones that matter to us (i.e., one’s we identify with) and that they are compatible with old identities (Iyer et al., 2009; Jetten, Iyer, Tsivrikos, & Young, 2008). Likewise, for social identity continuity to be beneficial for well-being, the new and the old identities need to be compatible. This is because tensions between past and present identities undermine the capacity for either to be a positive resource and support base (Jetten et al., 2014). This is relevant for older migrants, because if their new identities (e.g., host culture identity) are incompatible with their old identities (e.g., ethnic cultural groups), the positive association between multiple identities and well-being will be compromised—that is, social identity continuity will be disrupted and social identity gain will not be associated with enhanced social support.

### SIMIC and acculturation models

There are some interesting parallels between SIMIC pathways of identity continuity and gain and the observation in the literature that integration acculturation strategies are associated with higher well-being. In line with SIMIC, individuals who are well connected with their ethno-cultural or “old” identity while also taking on the new identity of the host culture experience not only social identity gain, but also a sense of social identity continuity, because the old identity remains alive and important in the new context while informing new social connections in the host culture. The integration orientation that results from such social identity continuity (i.e., maintenance of home culture) and social identity gain (i.e., contact with host culture) has been found to be related to lower levels of acculturative stress and higher levels of well-being (Berry, 1997).

The beneficial effects of belonging to multiple social groups (Iyer et al., 2009) might also contribute to understanding enhanced well-being with integration. The latter may be due to the fact that individuals who benefit from both identity maintenance and gain (i.e., through maintaining links with their identity of origin and taking on the new host identity) have more psychological resources at their disposal than those who are only connected to one cultural group (e.g., because the latter have adopted a separation or an assimilation acculturation strategy; Berry, 2017). As Berry states: “The associations afforded by these multiple social and cultural engagements may well offer the route to success in plural societies”.

### The current study

Previous research acknowledges that ageing in a foreign country away from one’s country of origin involves a major life transition, in which changes in various identities play a key role in adjustment. However, to date little is known about the particular processes that affect the migration experience of older migrants. Drawing on evidence from the recent social identity theorizing on social identity change and well-being, and the acculturation literature (e.g., Berry, 1997) more generally, we examine the experiences of older people who have migrated to Australia to better understand their influence in helping them to age in a foreign land.

Our research questions were developed to explore whether SIMIC processes are reflected in the experiences of older adults who have migrated to Australia. Specifically, we explore whether themes relating to identity continuity and discontinuity (RQ1), identity gain (RQ2), and identity compatibility (RQ3) describe and capture participants’ migration experiences. We chose to examine these research questions using qualitative methodology that enabled us to draw on people’s experiences of migration. Moreover, a qualitative approach allowed us to examine in more depth for the first time whether pathways identified in SIMIC would connect with and map onto the way that migrants themselves chose to narrate their migration experiences.

## Method

### Research context

This study was part of a larger program of research aimed at better understanding how engagement in social activities contributes to the well-being of people ageing in a foreign land. Participants were clients of an independent agency which provides multicultural social activities that aim to better link people with their ethnic cultures and the host culture more generally.

### Participants

Thirty-nine clients of the agency, who were all migrants to Australia, expressed an interest in participating in the study, and 29 (74.4%) agreed to be interviewed. Participants belonged to one of nine cultural groups (Chinese, Croatian, Dutch, El Salvadorian, German, Polish, South African with Indian origins, Spanish and Vietnamese). The majority of participants were female (*n* = 22, 75.9%) and between the ages of 66 and 92 years (average age = 74.41, *SD* = 6.39). The largest cultural group in the sample was Vietnamese (*n* = 12) followed by Chinese (*n* = 4) and Dutch (*n* = 4). The earliest arrival to Australia was in 1951, with the most recent in 2014, and the average length of time living in Australia was 34.29 years (*SD* = 15.94). Those coming from Germany and Poland were the earliest arrivals, while those from mainland China were the most recent. Participants had been clients of the agency for a period of 1 to 14 years, with the average length of time with the service being 4.79 years (*SD* = 3.33).

### Interview

The study involved one-on-one, semi-structured, in-depth interviews that lasted on average just under one hour. Participants were asked about (a) their experiences of leaving their country of origin and coming to Australia, (b) their current experiences living in Australia, (c) their attachment to their country of origin, (d) their feelings of cultural identity, (e) the social relationships they were involved in, (f) their social connections both in Australia and in their country of origin, (g) their views on the benefits (or detriments) of older people migrating to Australia, and (h) their experiences attending the social activity days organized by the agency. The topics were not presented in any particular order, and instead arose naturally during the course of the interview. Here, we focus on responses related to identity change (i.e., social identity continuity and gain and social identity incompatibility), along with narratives that speak to social connectedness and isolation.

To further contextualize people’s experiences, and take on board the wider literature on social isolation and migration, the interview included questions that required a quantitative answer. For these, participants were asked to rate their perceptions of quality of life and social isolation, both before and after arriving in Australia. Response options were presented on a scale from 1 (*very low/not at all*) to 10 (*very high/totally*).

### Procedure

The study received ethical clearance from The University of Queensland’s Human Research Ethics Committee (approval: 2,016,001,115). To test the appropriateness of the questions, a pilot study was conducted with six volunteers from different cultural backgrounds. Interviews for the main study were conducted between late November 2016 and early February 2017. Clients expressing an interest in participating in the study were informed that their involvement was voluntary and that the data they provided would not be linked to any personally identifying information. Participants provided their written informed consent prior to the interview. For those who asked to be interviewed in their ethnic language, the information and consent forms were translated orally into their ethnic language. All interviews were conducted face-to-face, and in the majority of cases (*n* = 21, 72.4%) interviews took place in the participant’s home. Eight interviews were conducted in a private room at the agency’s activity centre. Participants were given the choice to be interviewed in their native language, and six interviewers were trained to conduct these interviews in languages other than English. Participants were offered a $20 shopping voucher as compensation for their time and effort. The interviews were digitally recorded with consent from the participant and transcribed verbatim (those conducted in another language were transcribed and translated into English).

### Analytical strategy

A thematic analysis was conducted, using a combination of inductive (i.e., data driven) and deductive (i.e., theory driven) approaches. The thematic analysis followed the five-phase procedure described by Braun and Clarke (2006, 2012)). In the first *familiarization phase*, coders read transcribed interviews from start to finish. In a second *generating initial codes* phase, coders highlighted relevant sentences in the transcript and provided them with a label to identify a feature of the data. This step was repeated until all phrases were coded and until no new theoretically relevant themes could be identified. In phase 3, codes that appeared to describe a meaningful pattern in the data were grouped (*searching for themes* phase). To assist this process, relevant phrases were copied and pasted, and grouped together as clusters in a thematic analysis table. In a fourth *reviewing of potential themes* phase, themes in the thematic analysis table were reviewed in relation to the coded data and the entire data set. During this stage some themes were combined to form broader themes and, in other cases, themes were split into more specific sub-themes. Themes were then reviewed to ensure they captured the most important elements of the data set. In a fifth and final *defining and naming themes* phase, coders moved back and forth between data sets until theme content best reflected the semantic and latent conceptualizations conveyed in the data. Themes were also labelled during this phase.

**Table I.** Differences and similarities in experiences of the most and least socially isolated participants.

Highest Social Isolation	Lowest Social Isolation
**Differences in experiences**	
***Low social identity continuity***	***High social identity continuity***
Low sense of connection with country of origin	High maintenance of connection with country of origin
*“Friends, I have no friends [in Spain] because I’ve been too long here. All the family, [are] not like me.” (#35, male, age 78)*	*“When I’m in Holland I’m there, I’m happy and I see everyone…So when I’m in Holland all the family and the neighbours and everything, and people I’ve gone to school with, I still contact these people” (#12, female, age 82)*
Family support limited	High family support
*“All my children go to work. Nobody can take care of me. So I try my best, and always try to do things by myself.” (#19, female, age 71)*	*“I have a good family. My grandchildren always put a note ‘Welcome home’ at the door when I get out or away. When I return, I will first see it; it warms my heart, very happy. Our relationship is very close.” (#4, female, age 74)*
***Low social identity gain***	***High social identity gain***
High perceived cultural differences and discrimination	Cultural differences are emphasized to lesser extent
*“…they [children] grow up here in this society, and they don’t think the way Vietnamese do. Our Vietnamese people have our own good traditions, but they don’t value those traditions.” (#19, female, age 71)*	*“But this [Australia] is my second home, you know. So I’ve really become accustomed to it…And we had to get used to the food here. First time I had some prawns, I thought they were just striped carrots. Because I’d never seen prawns before…you know but it was great.” (#7, female, age 66)*
Concern over English language barrier	High English fluency or does not perceive low English proficiency as a barrier.
*“…over there I was able to cope better because we could speak the same language…. I could have learned English but I can’t when I am tired. Now I see how much I missed out on…That’s why I lost out on friendships here.” (#8, male, age 90)*	*“Many old people first were very enthusiastic about learning English, but soon gave up. I am different. I see learning English as my interest…Learning English is part of my life now.” (#4, female, age 74)*
Minimal contact with broader society	Mixes with different cultural groups, including Australian groups
*“I actually realise I have very little connection with Australian people now. Hmmm. I don’t know how that came but I really have very little.” (#6, female, age 83)*	*“What I like is all different nations. And I find it interesting to talk to people. I don’t tell that “That is strange, or that is funny”. I find them interesting. (#12, female, age 82)*
Few social connections	Socially well connected
*“But when he [husband] passed suddenly away, I felt very isolated…Well since my husband passed away its, well it’s not like I’m connected with a lot of people.” (#6, female, age, 83)*	*“But I’ve got a good lot of friends now. We go for coffee in the morning…I keep in touch with about three of them [previous work colleagues] and they come and you know we have lunch together and all that and I’ve got these friends, Filipino friends, there’s about eight of them.” (#39, female, age 72)*
**Similarities in experiences** Socially connected with own ethnic cultural groups Adherence to ethnic customs and food Experience of status loss Experience of physical health challenges	

Highest social isolation (*n* = 5) and lowest social isolation (*n* = 5). Categorizations are based on quantitative social isolation scores and self-reported qualitative interview data.

Three researchers were involved in the coding of the data (two of whom had also been involved in data collection). Two of these coders compared their coding after having analyzed six interviews involving participants from different ethnic groups. The coders reached close to 90% agreement, with complete consensus obtained after discussion. While coding continued, a third researcher, who was independent of the data collection process, was asked to analyze a subset of nine interviews from various cultural groups. A meeting was held after three of the nine interviews had been coded, and this revealed 85% agreement between coders. After discussion, a few new themes were identified and others were collapsed to form broader primary themes with more specific sub-themes. The independent researcher then coded the remaining seven interviews in the sub-set. In a subsequent meeting, no new themes were identified, but approximately 5% of phrases had been placed under different themes due their overlapping content. After discussion, full consensus was reached in relation to which themes and sub-themes best captured the content of these phrases.

## Results

### The study context

Participants reported a number of reasons for leaving their country of origin. Some felt they had no future in their country of origin in the aftermath of war (e.g., WWII, Vietnam war, Indonesia after the Japanese army withdrew) or as a result of a volatile political situation and persecution (e.g., Apartheid in South-Africa, political unrest and civil war in El Salvador). Others migrated to reunite with family, to be with their past or present Australian partner, because of work opportunities in Australia, or because they were looking for a life-change.

On average, participants rated their quality of life in Australia as higher (*M* = 8.22, *SD* = 1.58) than in their country of origin (*M* = 5.70, *SD* = 2.52). Moreover, the difference between quality of life prior to coming to Australia and while currently living in Australia was larger among those who reported migrating due to persecution in their country of origin (*n* = 15). For this group, the average quality of life score for their country of origin was 5 out of 10 (*M* = 5.00, *SD* = 2.93), while for Australia it was close to 9 out of 10 (*M* = 8.80, *SD* = 1.42), a difference of nearly 4 scale points. The difference between these scores was smaller for those who migrated for family or economic reasons (*n* = 12), with less than a 1 scale point difference between their experience of quality of life in their country of origin (*M* = 6.58, *SD* = 1.61) and their quality of life in Australia (*M* = 7.50, *SD* = 1.51). Eight participants did not provide a clear response to the social isolation question and so their ratings were excluded from analysis. While scores for social isolation were generally low, participants, on average, reported experiencing slightly more social isolation living in Australia (*M* = 3.10, *SD* = 2.05) compared with their country of origin (*M* = 1.86, *SD* = 1.18).

### Leaving the country of origin and moving to a new country

We examined participants’ narratives about their migration experiences relating to leaving their country of origin and moving to Australia. In these we were particularly interested in whether their experiences reflected social identity continuity and discontinuity (RQ1). Relevant data were highlighted three subthemes (1) the recognition and acknowledgement of loss, (2) nostalgia and longing for the country of origin, and (3) a loss of social status (i.e., pre-migration social status and professional identity). We will elaborate on these subthemes in turn.

**(1) The recognition of loss**. Many participants mentioned that the period immediately after arriving in Australia had been difficult, and some connected this with the disruption and discontinuity of their former social life. This is well captured by a Vietnamese immigrant who compared her life in Australia to her life back in Vietnam: “*I was sad. I was so used to life in Vietnam.”* (#15, female, age 76).

Others mentioned that the distance and expenses associated with travelling to their home country had made it hard for them to stay in touch with their loved ones back home This made it harder for them to remain connected to their past lives and social networks. For instance, a Dutch migrant mentioned:

*“…I said to [husband] “I have to go to Holland to see my mother” and he said “No, we haven’t got money for that. You have to wait until my parents are 50 years married and we might be able to go”. `And I said “But that would be too late”* (#14, female, age 78)

**(2) Nostalgia and longing for the country of origin**. For some, the experience of migration was associated with a nostalgic longing for the past and the country of origin. This sense of nostalgia emerged when thinking about the music, climate, and landscape in their country of origin:

*“Like yesterday all the [German] Christmas songs. Snow. I love snow. When I went [to Germany] I always went in winter. I can’t stand this [Queensland] weather…forests makes me homesick.”* (#5, female, age 80)

**(3) Loss of social status**. Participants also described experiencing a loss of social status and/or professional identity through the migration. Some noted that they had had higher social status in their country of origin and felt a profound sense of discontinuity as a result of status loss. Others felt that the experience of migrating had meant they had lost economic status because they had not been able to gain skilled employment in Australia. This is reflected in the following examples.

*“I mean we had a unit. We had a beautiful unit in Poland. And when we got thrown out [due to Nazi occupation], we got thrown out. When we came here we didn’t have that. We just started from the beginning*.” (#10, female, age 85)

*“I am a hairdresser. But I haven’t worked in Australia, because I don’t speak English very well and it is very difficult for me to work like this. And so I got work in a kindergarten, and continued in Canberra… And I was working in a factory in Ipswich. A factory that made underwear…”* (#37, female, age 81)

One participant described status loss not as something she had experienced personally, but felt it was a common experience for many of her fellow migrants from China:

*“They [some Chinese migrants] felt that they were of high status in China; many people held respect for them. They really need to adjust. When you came to Australia, you have to leave other things like status behind.”* (#4, female, age 74)

It was also clear that participant’s narratives related to experiences of continuity, that people made substantial efforts to *maintain* valued social identities (as SIMICs *social identity continuity pathway* suggests). Several approaches were used to achieve this sense of continuity.

**(1) Staying connected with those in the country of origin**. Despite the fact that many of our participants migrated to Australia at a time when it would be difficult to remain in close contact with family and old group memberships in the country of origin (i.e., in the 1950 and 60s), migrants emphasized that they still had been able to stay in touch with people that mattered to them in the country of origin. This is illustrated in the following example:

*“We were kind of a clover, three girlfriends and myself in Germany. We knew each other from primary school and we are still in close contact, very close contact. Every time I go over there or organize a class reunion. So with one I would have almost every second day emailed.”* (#5, female, age 80)

**(2) Engaging in cultural activities**. Many participants mentioned that they engaged in cultural activities that connected them to their country of origin (such as preparing food together with other people).

*“It’s just the German group. We meet once a month. Last week we went all to Adam’s, the Butcher. It’s in Carol Park. A German butcher. So we go there every May and every November. So I can buy my German meat and sausages and all that…Friday and Saturday night they have a dance but it’s usually just for everyone.”* (#7, female, age 66)

*“On Thursdays, I’m with the Women group in the Inala Hub…The Hub organizes meetings for women from Vietnam and other cultures. They teach us to do crafting, cooking, etc.”* (#25, female, age 77)

**(3) Maintaining hobbies and activities**. Participants also described keeping up with activities and hobbies they started in their country of origin, as a way of staying connected to their home country. For example, one participant emphasized that playing the piano allowed her to connect with that past:

*“And I was brought up in a very musical family […] and that was a big part of my life… So then [in Australia] I cleaned here and there and I found a lady […] and she had been a music teacher and had a piano there. So even though she couldn’t walk anymore and so on, she taught me. She said ‘If you make a bit of time, I’ll teach you piano’. So she did. She told me what to do and very soon I learned piano and she said ‘You have a real gift for piano’. So that was great for me…”* (#14, female, age 78)

**(4) Connecting via media**. Others maintained a sense of connectedness with their country of origin through via media:

“So, we don’t feel like Australian. We installed a big device to allow us to watch

*TV programs from China, HK and Macau.”* (#2, male, age 74)

*“Someone said “Oh, it’s great to have the satellite and you can watch the Dutch programs and everything”. We watch America and we watch Holland. And regularly keep track of what’s going on in Holland now.”* (#14, female, age 78)

**(5) Connecting through volunteering**. Finally, a sense of connectedness to the pre-migration country and identity was also nurtured by spending significant amounts of time with other people from their country of origin. Interestingly, much of this contact involved helping others and volunteering in groups that provided a service for migrants from their country of origin. This provided them not only with an opportunity to help with some of the barriers that the migrants themselves might have faced, such as language difficulties, but also to help others with issues of isolation in older age. This is evident in an account from a German participant:

*“Like last time I had a contact to go and visit an old age home with a German lady that couldn’t speak much English, so they asked me to come in and translate for her so she could sign some documents for consent. She couldn’t understand anything. So I sent in there and talked to her. So I’m trying to stay in touch with them and give her some German books. Like little booklets, love stories and things. And German cities and magazines so she can have at least something because she is all on her own in there.”* (#7, female, age 66)

Together, these findings highlight the important role that continuity plays to a person’s experience of adjustment to migration. In people’s narratives, a sense of social identity (dis)continuity (1) was acknowledged, (2) triggered nostalgia, and (3) was reflected in experiences of loss in social and professional status. It was also clear that themes that related to experiences of social identity continuity were associated with positive emotions and social identity discontinuity with negative emotions. These accounts also showed that participants strived to maintain a sense of connectedness (enabling social identity continuity) with their home country by (1) staying in contact with people from their home country, (2) engaging in cultural activities (in particular preparing food from their country of origin), (3) still engaging in activities and hobbies that they started in their country of origin, (4) keeping in touch with their home country through the media, and (5) engaging in volunteering activities that involved helping others from the same country of origin (often with challenges that related to their migratory experience).

### Social connectedness after arrival in the new country

A common theme that emerged from the interviews was people’s experiences of meeting new people and joining groups when they had just arrived in Australia—something that links with SIMICs social identity gain pathway (RQ2). Consistent with the notion that gaining membership in groups and social networks that form the basis of new social identities facilitates adjustment, many migrants spontaneously mentioned that they sought out social groups—mostly groups that comprised people from the same cultural, religious and language background—when they had just arrived in Australia. Clearly, this had helped them to start their new life. For example:

*“It has been very good. Because when we came here, the Church, the Reform Church [started by Dutch migrants]. They had a church in Toowong. But they just started a small group in Inala. And the people were so good. Every Sunday we were asked somewhere.”* (#14, female, age 78)

*“The first time we went in the Dutch Club… And it was just like getting into Heaven. Because all these people that we knew from the other churches and never met anymore, and there they all were, heh? All Dutch migrants. So it was great to meet each other again.”* (#14, female, age 78)

When participants were asked about their current social connections and how this contributed to their well-being, they talked about taking part in spontaneous or organized group activities with other migrants from the same country of origin. Many of the activities in these groups focused on cooking or engaging in activities that celebrated their home culture. It also allowed them to speak in their native language. Interestingly too, there was clear evidence that adjustment after migration depended not so much on whether people had belonged to multiple social groups and had many social ties before they migrated, but whether they had quickly formed new relationships and joined groups after arrival in Australia. For example:

*“I have close friends. When we meet, we meet at one of the houses, and make cakes or Vietnamese specialties together..”* (#20, female, age 67)

*“But you know. Spanish is my language. And I understand everything they talk about. And I can answer or leave it. That’s why I come [to Spanish group]. Plus not to be alone at home, you know.”* (#35, male, age 78)

*“I’ve got everything at the German Club…I’ve been going to the club since we got here in Australia, so I know everyone there…”* (#7, female, age 66)

*“I also go to the church. Every weekend and Fridays too. I meet my [Vietnamese] friends, and relatives when I go to the church, and talk to them.”* (#15, female, age 76)

These data suggest that joining new groups immediately after migration provided a new sense of grounding and belonging that helped people to start a new life in Australia and this seemed to be associated with feeling supported and, by extension, better adjusted. In particular, and consistent with SIMIC, there was some evidence that joining new groups following an important life transition such as migration can protect and even reverse the negative effects of change. This is because, to the extent that the social support derived from these group memberships (and the psychological resources they provide) are likely to make the experience of entering a new phase in life a less stressful experience (see Haslam, Jetten, Haslam, & Postmes, 2009). Speaking to issues of identity compatibility, it also emerged that migrants were selective in the groups that they joined and they preferred groups that were composed of people from the same ethnic or national background.

### Social identity compatibility and social isolation

To further understand people’s experiences, we examined the narratives of those who are most and least isolated in more detail. Specifically, drawing on the earlier quantitative ratings of social isolation we examined whether people’s experiences of continuity, gain and identity compatibility differed as a function of their perceptions of isolation after migrating.

To identify the most isolated individuals, we explored the nature of comments that participants had made on their feelings of being isolated (directly or indirectly) at different times during the interview. To identify the least isolated individuals in our sample, we carefully examined responses by participants who had frequently mentioned their strong connections with people. This process identified five individuals who experienced the most isolation and five individuals who experienced the least isolation in the sample. Importantly, these participants included migrants from different cultures, indicating that social isolation was not unique to any particular nationality. After identifying these participants, we also examined their ratings of social isolation on a 10-point scale described earlier. This comparison confirmed our classification and selection, with scores for social isolation in Australia higher for the highest isolation group (*M* = 5.75, *SD* = 0.96), compared with the lowest isolation group (*M* = 2.00, *SD* = 1.41).

Table I provides an overview of key similarities and differences in experiences described by migrants in these two groups. For each key difference, an example is provided for illustration. Note that the differences identified are not necessarily related to every person in either of the two groups. For the most isolated migrants, we found little evidence of social identity continuity between pre-migration and post-migration social connections. What was notable in these descriptions was the low sense of connection that people felt with their country of origin and they reported that family support was limited. However, for the least socially isolated individuals, social identity continuity appeared to be relatively high: they highlighted valuing contact with people in their country of origin and making an effort to keep those relationships alive. These individuals also reported receiving greater family support more generally.

People with greater social isolation felt they had joined few new groups since arriving in Australia—describing greater cultural differences, more concerns about English language barriers, minimal contact with broader society, and few social connections. In contrast, those who were least socially isolated reported fewer barriers to becoming socially connected in Australia (e.g., not seeing cultural differences, having less concern about lack of English proficiency), and they described developing new connections with multiple social groups not only when they arrived in Australia but also in later life (e.g., reports of high social connectedness with both their ethnic community and with Australians and Australian groups). Despite these differences, there were also a number of similar experiences described by those who reported lower and higher levels of social isolation . In particular, most participants from both groups reported being socially connected with their own ethnic group, adhering to ethnic customs and eating food from their culture. They also shared negative experiences relating to status loss as a result of migrating and age-related physical challenges.

Overall, despite the similarities in terms of the challenges that both the least and most socially isolated faced, there were also some differences that could be connected to the two pathways to understanding adjustment to life change that SIMIC predicts: social identity continuity and social identity gain. Interestingly, it is here where themes relating to compatibility became most apparent—the most isolated individuals pointed to the differences between their pre-migration and post-migration identity (e.g., in terms of culture) and difficulties in managing the challenges posed by their new environment (e.g., speaking English and the difficulty of connecting). In line with SIMIC, these were related to the difficulties maintaining social identity continuity and that stood in the way of social identity gain. In contrast, those who experienced lower levels of social isolation appeared to focus less on differences, difficulties and challenges post-migration (i.e., higher identity compatibility) and they were more likely to describe their post-migration experiences in terms of social identity continuity and gain.

## Discussion

The present findings allow a more elaborated understanding of older migrants’ experiences in the host cultural context, and these we used these to explore the relevance of processes identified in SIMIC to people’s experience of migration. We found evidence that the narratives of older immigrants did indeed capture aspects of the two key pathways identified within SIMIC—the social identity continuity pathway and the social identity gain pathway. In particular, in relation to identity continuity (Haslam et al., 2008; Jetten & Hutchison, 2011; Jetten & Wohl, 2012; Sani, 2010), we found that participants connected their migration experiences to challenges relating to the maintenance of connectedness between their pre-migration selves and post-migration selves (RQ1). Specifically, social identity (dis)continuity was acknowledged, triggered nostalgia, and emerged in accounts of social status loss. Participants also made efforts to maintain a sense of connectedness between their past and current identity. In addition to evidence that this was achieved by maintaining connectedness with others (e.g., by staying in touch with friends and family in their country of origin or by connecting with other migrants from the home country), the data also show that people engaged in attempts to ensure cultural connectedness and continuity (e.g., by engaging in cultural activities or hobbies connecting them to their country of origin, or by accessing media from their home country). Regardless of the way in which social identity continuity was enabled, extending valued old identities in a new cultural context appeared to strengthen participants’ sense of belonging and social connectedness, and this contributed to well-being.

Furthermore, in line with previous work providing evidence for the importance of the social identity gain pathway in times of change (Cruwys et al., 2014), it appeared that joining new groups was beneficial in helping people to adjust to the new context (RQ2). Gaining new group memberships enhanced connectedness and this, at least partly, countered the negative effect of loss of old identities by enabling access to a larger social support network.

Finally, when considering those who were most socially isolated and those who were least socially isolated, we also found some evidence that social connectedness (and belonging to multiple social networks in particular) immediately after arrival in Australia facilitated adjustment. Those who were currently least socially isolated were also more likely to quickly form new social connections when they had arrived in Australia. It appears that this was associated with an increase in available social support resources to cope with cultural transition. Also noteworthy in the context of interrogating SIMIC, is the findings that it was not so much multiple group memberships before the change, but whether people quickly developed a social network after arrival that predicted well-being and adjustment. This is perhaps not surprising given the fact that, due to the distance, many participants in our sample were simply unable to stay in touch with close friends and family in their country of origin. This suggests that the inability to rely on old social connections in the new context (and the difficulty of maintaining social identity continuity through maintaining strong pre-migration social networks) may have made the social identity gain pathway particularly important in predicting adjustment and well-being for these older migrants.

Interestingly too, the comparison of experiences of the least and most socially isolated individuals also revealed some evidence for the importance of identity compatibility in adjustment (RQ3). In particular, those who were most isolated described themselves as being culturally different from the host culture and highlighted challenges associated with integration (e.g., English language difficulties). In line with SIMIC, this prevented them from developing a sense of continuity between pre-migration and post-migration identities and it stood in the way of gaining new social connections. Moreover, and in line with SIMIC, there was evidence that those who saw their old identities as compatible, rather than conflicting, with new identities reported higher well-being and adjustment (Iyer et al., 2008, 2009).

### Implications for theory

The findings, then, provide evidence for the pathways identified in the SIMIC model and extend its application to new contexts. That is, even though SIMIC pathways have been examined among people with acquired brain injury (Haslam et al., 2008; Jones et al., 2012) and students entering university (Iyer et al., 2009; Jetten et al., 2008), SIMIC has not yet been explored in the context of identity change that involves migration from one country to another. Moreover, even though SIMIC predictions had been examined in the context of identity change among older adults, these samples were limited to participants from Western cultures (e.g., Gleibs, Haslam, Haslam, & Jones, 2011; Haslam et al., 2010). Here, we show that SIMIC also helps to explain identity change related to migration from a range of countries, and that the model helps to understand adjustment and well-being among older migrants ageing in a foreign land.

Furthermore, our analysis connects in important ways with research on acculturation in predicting adjustment, enriching both models in important ways. In particular, Berry’s (Berry, 1997, 2001) model of acculturation is based on a distinction between two dimensions: (1) a preference for maintaining one’s cultural heritage, and (2) a preference for having contact and participating in the host community. It has been shown that integration acculturation strategies—whereby individuals maintain their cultural integrity but at the same time seek to take part in the host community—are associated with better adjustment. This is consistent with our findings, which suggest that the roots to adjustment and healthy ageing can be traced to the way that participants experienced the process of social identity change associated with migrating. We found that adjustment involved successful social identity change whereby (a) the old identity remained important and represented in the new context and (b) a process of “moving on” whereby new social identities were gained in Australia.

There are also some interesting parallels between SIMIC and Berry’s model of acculturation when it comes to predictors of poor adjustment. Berry’s model (Berry, 1997, 2001) suggests that two less adaptive strategies of acculturation involve separation (which occurs when migrants wish to maintain their heritage culture but lack interest in interacting with other cultures) or marginalization (an acculturation strategy where there is little interest in cultural maintenance and a lack of willingness to interact with other cultures). Our results showed that, in line with a separation acculturation orientation, those who showed the highest levels of social isolation appeared not to have gained many new social identities after arrival in Australia (either as a result of perceived discrimination and exclusion, or due to language barriers). Moreover, and in line with a marginalization acculturation orientation, these individuals also showed little social identity continuity: they did not feel particularly connected to their country of origin, and there was little sign of continuity between the past and present. These parallels between SIMIC and acculturation models are interesting and suggest that processes of social identity change and acculturation are intimately linked, whereby each highlights different aspects of the same coin that help us to understand adjustment and well-being among migrants.

Interestingly too, the qualitative analysis allowed for themes to emerge that we had not anticipated. In particular, we not only found evidence for the predicted theme that interacting with others from the same national background facilitated social identity continuity, it also emerged that such contact often took the form of helping those others with particular challenges they faced. Many offered their help to other migrants who experienced language barriers or were at risk of isolation—difficulties with which our participants were probably quite familiar. This is consistent with the social identity theory notion that shared social identity forms not only the basis for receiving and benefiting from sources of social support (Haslam, O’Brien, Jetten, Vormedal, & Penna, 2005), offering such social support appears to be equally, if not more, beneficial for wellbeing (Steffens, Jetten, Haslam, Cruwys, & Haslam, 2016). This finding is particularly interesting to follow up on, because it suggests that volunteering to help others from a similar background offers not only benefits in terms of the provision of support, it also has benefits for those offering the help because it provides a basis for social identity continuity.

The qualitative approach was a clear strength of this research. The qualitative analysis allowed us to shed light on a number of processes that would have been difficult to capture quantitatively. Notably, our analysis provided evidence that when explaining social isolation and well-being, participants spontaneously mentioned experiences that speak to broader themes of social connectedness, social identity continuity and gain. This is reassuring, and the validity of SIMIC is enhanced by the finding that processes that are central to SIMIC appear without much prompting in the language and narratives of our participants when asked about their migration experiences. It would be important to further examine these findings in other contexts, focusing on other migrant groups and different national contexts. Moreover, to overcome some of the limitations of this research due to our subjectivity, it would also be worthwhile for other research teams to explore examine these processes. This would enrich the analysis and it would provide insight in the generalizability of our findings.

## Conclusion

Research provides clear evidence that older migrants are vulnerable to social isolation as they age in a foreign land. By recognizing that adjustment to identity transitions such as migration involves identity change, we can extend our understanding of the process and use this knowledge to identify ways of helping those undergoing change to better adjust. In recognition that social groups are an important psychological resource, we found that social identity continuity and social identity gain were important pathways that have the capacity to protect the well-being for older adults adjusting to life changes associated with migration. Importantly, formal organizations that help to maintain connectedness to one’s ethnic roots (social identity continuity) while also providing opportunities for social interaction with the broader community (social identity gain) have an important role to play in enhancing the quality of life of older migrants (Cook, 2010). Identifying ways in which identity resources can be harnessed to realize their health-enhancing potential forms an important avenue for future efforts to facilitate ageing in a foreign land.
